# A significant *P* value: How phosphorus controls plant height

**DOI:** 10.1093/plcell/koad284

**Published:** 2023-11-07

**Authors:** Kutubuddin A Molla

**Affiliations:** Assistant Features Editor, The Plant Cell, American Society of Plant Biologists; ICAR-National Rice Research Institute, Cuttack 753006, India

Phosphorus plays key roles in photosynthesis, respiration, development, reproduction, and the biosynthesis of membranes and nucleic acids (Lambers 2022). Modern agriculture is highly dependent on inorganic phosphorus (Pi). Soil Pi deficiency induces a series of physiological changes and can affect plant architecture, including plant height. However, the molecular mechanism governing Pi-regulated plant height remains largely unknown. Taller rice plants tend to fall over in strong winds and heavy rains, a phenomenon known as lodging. Amid climate change and more extreme weather events, such as cyclones, typhoons, and heavy rains, crop lodging often leads to yield loss. Plant height is intricately linked with resistance to lodging and yield. During the Green Revolution, the improvement in yield for rice and wheat was attributed to changes in plant architecture, specifically a reduction in plant height linked to lodging resistance. Although semi-dwarf rice varieties have played a significant role in feeding the world over the last 50 years, shallow lowland and semi-deep ecologies demand rice varieties with tall heights and lodging resistance.

In this issue, **Tingting Wang and colleagues (Wang et al. 2023)** identified a Pi-dependent transcription factor, MYB110, that negatively regulates plant height and might provide an attractive strategy for breeding tall rice plants with lodging resistance and high yield (see Fig.). The authors found that *MYB110* is induced under Pi starvation and swiftly repressed upon Pi resupply. Interestingly, the knockout mutant *myb110* plants were significantly taller than wild type, irrespective of soil Pi status, and the Pi starvation-induced inhibition of plant height was largely compromised upon *MYB110* mutation. In contrast, growth in *MYB110* overexpression (*MYB110-Ox*) lines was inhibited in both high- and low-Pi conditions. These findings suggest that MYB110 acts as a Pi-dependent negative regulator of plant height in rice.

**Figure.**
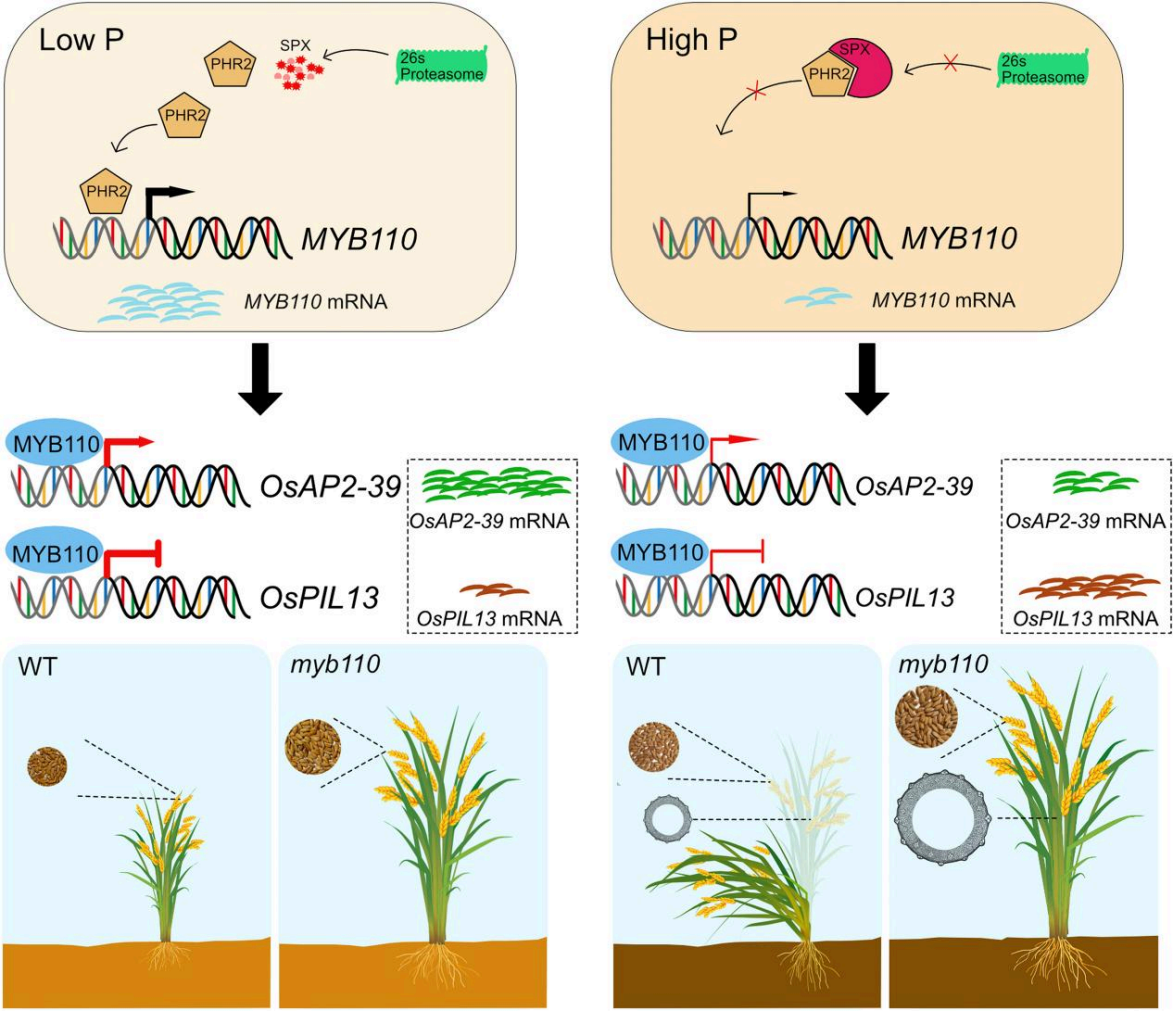
A proposed working model of Pi-dependent MYB110-mediated regulation of plant height, lodging resistance, and yield in rice. *MYB110* is a direct target of PHR2. MYB110 represses *OsPIL3* while activating *OsAP2-39* to regulate plant height in response to Pi supply. Knockout of *MYB110* increases plant height, lodging resistance, grain size, and yield compared with wild type. Adapted from Wang et al. (2023), Fig. 10.

The authors then focused on *PHOSPHATE STARVATION RESPONSE 2* (*PHR2*), a central regulator of plant Pi starvation signalling identified in earlier studies (Zhou et al. 2008; Liu et al. 2010). Overexpression of *OsPHR2* retarded growth regardless of Pi availability. Interestingly, the authors detected a PHR1 binding sequence (P1BS) in the promoter of *MYB110*, suggesting that MYB110 could be a direct target of PHR2 in regulating plant height. Expression analysis of *MYB110* in *phr2* mutant and *PHR2-Ox* lines suggested that PHR2 positively regulates *MYB110* expression. Multiple follow-up assays confirmed that PHR2 physically binds to the P1BS motifs in the *MYB110* promoter and that OsPHR2 functions upstream of *MYB110*.

To identify MYB110's direct downstream genetic targets, the authors analyzed the promoter sequences of genes differentially regulated in *myb110* mutants. This analysis and further assays demonstrated that *OsAP2-39* and *OsPIL13* are direct targets of MYB110. Previous work demonstrated that *OsAP2-39* regulates ABA/GA balance to control plant height, and *OsPIL3* regulates internode elongation in rice (Yaish et al. 2010; Todaka et al. 2012).

Surprisingly, the *myb110* as well as the *MYB110-Ox* lines showed increased lodging resistance compared to the wild type under high-Pi conditions. Both *myb110* and *MYB110-Ox* lines showed increased resistance to bending of stem internodes compared with wild type. However, *MYB110-Ox* lines exhibited increased lignin deposition in the stem, whereas *myb110* mutants showed decreased lignin deposition but increased thickness between the inner and outer radius of the internode cross-section and in sclerenchyma cell layers. Hence, lodging resistance was attributed to reduced height and elevated lignification in *MYB-Ox* lines but the increased stem internode thickness in the taller *myb110* mutants (see Fig.).

The wild-type and *MYB110-Ox* plants exhibited significant yield penalties under low-Pi conditions compared with high-Pi conditions. In contrast, *myb110* mutant lines showed significantly higher yield under both low- and high-Pi conditions compared with wild type. Analyzing 270 rice samples, the authors identified 2 haplotypes comprising 4 SNPs in the *MYB110* promoter and 1 in the ORF. Among the germplasm set, Indica cultivars were enriched with Hap1 and japonica cultivars with hap2. Genotypes with Hap2 showed higher MYB110 expression and shorter height compared with genotypes with Hap1.

Thus, the study by Wang et al. (2023) identified a distinct candidate gene that has the potential to increase plant height as well as increase lodging resistance and yield.

## Dive Curated Terms

The following phenotypic, genotypic, and functional terms are of significance to the work described in this paper:

MYB110 Gramene: AT3G29020
MYB110 Araport: AT3G29020

plant height Planteome: TO:0000207

PHR1 Gramene: AT4G28610
PHR1 Araport: AT4G28610

PHR2 Gramene: Os07g25710
PHR2 Araport: Os07g25710
